# Low-Intensity Extracorporeal Shock Wave Therapy (LI-ESWT) for Erectile Dysfunction in Patients Post-prostatectomy: A Systematic Review

**DOI:** 10.7759/cureus.93901

**Published:** 2025-10-05

**Authors:** Abhinav Singhal, Maanya Bhardwaj, Gaurika Bhardwaj, Keval M Patel

**Affiliations:** 1 Urology, University Hospitals Birmingham NHS Foundation Trust, Birmingham, GBR; 2 Urology, Cambridge University Hospitals NHS Foundation Trust, Cambridge, GBR; 3 Critical Care, Royal Marsden Hospital, London, GBR

**Keywords:** ed, erectile dysfunction, iief, impotence, li-eswt, low-intensity extracorporeal shock wave therapy, prostatectomy, radical prostatectomy, sexual function improvement, shock wave therapy

## Abstract

Erectile dysfunction (ED) is a common and distressing complication following radical prostatectomy, significantly affecting patients' quality of life. Low-intensity extracorporeal shock wave therapy (LI-ESWT) has emerged as a non-invasive modality aimed at enhancing erectile function through tissue regeneration and improved neovascularisation. This review aims to systematically evaluate the safety and efficacy of LI-ESWT in the treatment of ED in men following prostatectomy.

This systematic review was conducted following the Preferred Reporting Items for Systematic Reviews and Meta-Analyses (PRISMA) guidelines. A comprehensive literature search was performed across PubMed, PubMed Central (PMC), MEDLINE, Cochrane Library, and Scopus for studies published between January 2015 and July 2025. Eligible studies included randomised controlled trials (RCTs) and cohort studies evaluating LI-ESWT for post-prostatectomy ED using validated outcome measures such as the International Index of Erectile Function-5 (IIEF-5) and Erection Hardness Score (EHS). Risk of bias was assessed using the Cochrane Risk of Bias (RoB) 2.0 and the Newcastle-Ottawa Scale.

Ten studies, of which five were RCTs and five were cohort studies, involving a total of 760 patients met the inclusion criteria. LI-ESWT was associated with statistically significant improvements in erectile function across most studies, particularly when combined with phosphodiesterase-5 inhibitors (PDE5is). IIEF-5 scores improved in both three-monthly and 6-12-monthly follow-ups, and EHS ≥3 was commonly achieved in combination therapy cohorts. No serious adverse events were reported. However, variations in treatment protocols, energy settings, and follow-up durations limited comparability and precluded meta-analysis. The overall certainty of evidence was moderate for efficacy and high for safety.

LI-ESWT appears to be a safe and potentially effective treatment for ED following radical prostatectomy, particularly when initiated early and used alongside PDE5is. Despite encouraging results, heterogeneity among studies and treatment protocols, alongside methodological limitations, underscores the need for larger, high-quality RCTs to establish standardised protocols and confirm long-term benefits.

## Introduction and background

Erectile dysfunction (ED) is defined as the persistent inability to attain and maintain an erection sufficient for the satisfaction of both sexual partners [1,2]. Several factors are implicated in the causation of ED, such as vascular, neuronal, hormonal, and metabolic factors. Arterial dilatation, smooth muscle relaxation, and veno-occlusion within the penile corpora are prerequisites for normal erectile function [2,3]. ED is a frequent complication post-prostatectomy, with nearly all men experiencing some degree of dysfunction due to trauma and disruption to the cavernous nerves [4]. Erectile function is reportedly regained in 30-60% of patients one year post-surgery [3,4]. ED may result in various psychosocial and physical health complications, such as lack of sexual confidence, low self-esteem, depression, and interpersonal and relationship difficulties [5]. There is also an association with cardiovascular disease, stroke, and peripheral arterial disease, as these conditions share the same pathophysiological mechanisms and risk factors as ED [5,6]. Underlying mechanisms include endothelial dysfunction, chronic inflammation, vascular structural alterations, dysfunctional nitrous oxide pathways, and abnormal peripheral sympathetic activity [3,6]. 

Low-intensity extracorporeal shock wave therapy (LI-ESWT) has recently emerged as a non-invasive treatment option for ED [7]. Unlike pharmacological treatments such as phosphodiesterase-5 inhibitors (PDE5is), which provide temporary symptom relief, LI-ESWT is proposed to induce biological changes that may restore erectile function. LI-ESWT is proposed to improve erectile function by stimulating neovascularisation, enhancing blood flow, and promoting regeneration in penile tissues [7,8]. Shock waves create mechanical stress in tissues, stimulating the release of angiogenic factors such as vascular endothelial growth factor and endothelial nitric oxide synthase [5,8]. This promotes the formation of new blood vessels and improves penile microcirculation, enhancing oxygenation and blood flow [6,7]. Furthermore, LI-ESWT induces microtrauma, which activates endogenous repair pathways for tissue regeneration and remodeling [3,5-8]. Post-surgical ED is largely driven by nerve injury and reduced penile perfusion; LI-ESWT addresses both by promoting vascular and neurogenic recovery. 

Preliminary studies suggest that LI-ESWT may offer long-term therapeutic benefits, especially for men with vasculogenic ED [8]. However, clinical evidence remains varied in quality and findings. This systematic review aims to evaluate and summarise current evidence on the efficacy and safety of LI-ESWT as a treatment modality for post-prostatectomy ED. 

## Review

Methods

This systematic review was conducted in accordance with the Preferred Reporting Items for Systematic Reviews and Meta-Analyses (PRISMA) guidelines [9]. Studies were selected according to the inclusion and exclusion criteria specified in Table 1. 

**Table 1:** Exclusion and inclusion criteria for the studies ED: erectile dysfunction; IIEF: International Index of Erectile Function; EHS: Erection Hardness Score

	Inclusion	Exclusion
Population	Adult men (≥18 years) diagnosed with ED post-prostatectomy	Women, adult men with ED due to any other aetiology, adolescents or children (<18 years), animal or in vitro studies
Study design	Randomised controlled trials and prospective or retrospective cohort studies	Case reports, case series, case-control studies, systematic reviews, and meta-analyses
Language	Papers in English	Non-English studies
Timeframe	Publications between 2015 and 2025	Studies not in this timeframe
Outcomes	Clinical outcomes related to erectile function, such as IIEF scores, EHS, and other validated erectile function measures	Studies not reporting on erectile function or relevant clinical outcomes

**Figure 1:**
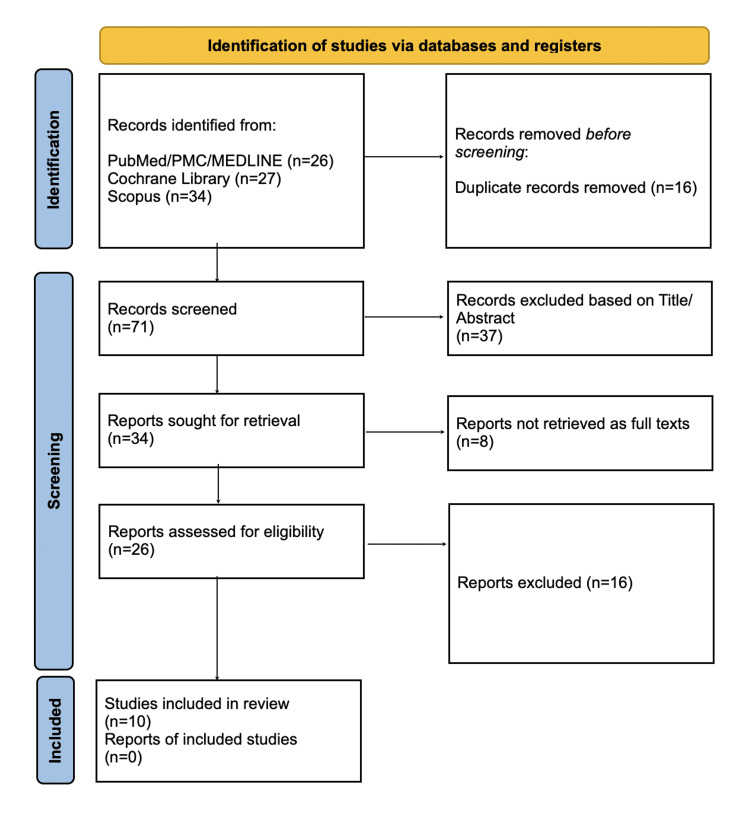
PRISMA flow diagram for selecting studies for inclusion in the review PRISMA: Preferred Reporting Items for Systematic Reviews and Meta-Analyses; PMC: PubMed Central

A comprehensive search was conducted in the following electronic databases: PubMed, PubMed Central (PMC), MEDLINE, Cochrane Library, and Scopus. Articles published from January 1, 2015, to July 1, 2025, were included in the search. A structured search strategy was developed using keywords and Medical Subject Headings (MeSH) terms. The search terms included combinations of the following: "Erectile Dysfunction", "ED", "Impotence", "Low-intensity extracorporeal shock wave therapy", "LI-ESWT", "Shockwave therapy", "IIEF-5", and "Sexual function improvement". Search strategies used to identify relevant papers in the databases are available in the Appendices. Titles and abstracts were screened independently by three reviewers using the defined inclusion and exclusion criteria. Full texts for potentially eligible studies were retrieved and assessed for final inclusion. Figure 1 shows the PRISMA flow diagram, which outlines the process used for selecting studies for the review [9]. Data was extracted independently by two reviewers using a standardised data extraction form; discrepancies were resolved by discussion and, where necessary, adjudication by a third reviewer. The data collected included study characteristics, population details, intervention details, outcome measures, key findings, and conclusions. The methodological quality of the included studies was assessed using the Cochrane Risk of Bias (RoB) 2.0 tool [10] for randomised controlled trials (RCTs) and the Newcastle-Ottawa Scale (NOS) [11] for cohort studies. Each domain was rated as low, moderate, or high risk of bias. Discrepancies were resolved by consensus and discussion among three reviewers. 

Due to heterogeneity in study design, interventions, comparators, and outcome reporting, we prespecified a narrative synthesis rather than a quantitative meta-analysis. The included studies varied substantially in type (RCTs, cohort studies), treatment protocols, and outcome measures (International Index of Erectile Function-5 (IIEF-5), Erection Hardness Score (EHS), penile Doppler). Furthermore, outcome measures were inconsistently reported, with many studies lacking standardised effect sizes or variance data, precluding statistical pooling. 

Results

PRISMA guidelines were utilised for the study selection process, and the flow diagram is shown in Figure 1. A total of 87 studies were identified using the databases (26 from PubMed/PMC/MEDLINE, 27 from Cochrane Library, and 34 from Scopus), of which 71 remained after the removal of duplicates. After screening the titles and abstracts, 10 full-text articles were evaluated based on the inclusion criteria specified in Table 1. As a result, 10 papers with a total of 760 patients were included in the final analysis, with a summary of their characteristics outlined in Table 2. Five studies were RCTs, three were retrospective cohort studies, and two were prospective cohort studies. Studies were included if they evaluated the effects of LI-ESWT on erectile function recovery post-prostatectomy and reported measurable outcomes such as the IIEF-5 or EHS. 

**Table 2:** Characteristics of the included studies LI-ESWT: low-intensity extracorporeal shock wave therapy; RCT: randomised controlled trial; RCS: retrospective cohort study; PCS: prospective cohort study; SHIM: Sexual Health Inventory for Men; IIEF-5: International Index of Erectile Function-5; LSTC-ED: Linear Shockwave Tissue Coverage-Erectile Dysfunction; EHS: Erection Hardness Score; PDE5i: phosphodiesterase-5 inhibitor; LI-LiESWT: linear low-intensity extracorporeal shock wave therapy; RARP: robot-assisted radical prostatectomy

Study	Study design	Key findings	Sample size		Protocol of LI-ESWT treatment				
			Control group	LI-ESWT group	No. of treatments per week	No. of sites	Total duration of treatment (weeks)	Follow-up (months)	Outcome measures
Motil et al. [12]	RCT	LI-LiESWT (LSTC-ED® technique) significantly improved IIEF-5 scores at 2 and 3 months; benefits persisted at 12 months	16	16	1	Penile shaft (exact sites not specified)	4	6 and 12	IIEF-5
Baccaglini et al. [13]	RCT	Weekly LI-ESWT improved erectile function vs. baseline; well-tolerated	41	36	1	4	8	Not explicitly specified	IIEF-5, EHS
Pedersen et al. [14]	RCT	Sham-controlled RCT showed significant improvement in IIEF-5 and EHS in the LI-ESWT group	31	33	1	6	5	3	IIEF-5, EHS
Jang et al. [15]	RCT	LI-ESWT+tadalafil showed higher EHS ≥3 and improved erectile function at 6 months vs. tadalafil alone	39	41	2	5	6	6	IIEF-5, EHS
Kohada et al. [16]	RCS	LI-ESWT showed superior long-term erectile function recovery post-RARP compared to PDE5i-only and untreated groups	16	139	2	5	6	12	IIEF-5, SHIM score
Frey et al. [17]	PCS	Open-label prospective cohort showed IIEF-5 and EHS improvements at 12 months; no control group	No control group	16	2	5	6	12	IIEF-5, EHS
Karakose and Yitgin [18]	RCS	LI-ESWT+tadalafil improved erectile function and continence vs. tadalafil alone	32	34	2	5	6	3, 6, 12	IIEF-5
Sholan et al. [19]	PCS	LI-ESWT+tadalafil yielded greater erectile function recovery than LI-ESWT monotherapy	18	20	2	5	6	12	IIEF-5, EHS
Inoue et al. [20]	RCS	LI-ESWT resulted in improved recovery of erectile function post-RARP compared to non-rehabilitation patients	16	178	1	5	6	3, 6, 9, 12	IIEF-5, penile Doppler
Ladegaard et al. [21]	RCT	RCT demonstrated statistically significant improvement in IIEF-5 and EHS vs. placebo	18	20	1	6	5	1, 3	IIEF-5, EHS

Across studies, LI-ESWT was delivered using similar energy settings (typically 0.09 mJ/mm²) over 6-12 sessions. In the RCT by Baccaglini et al. [13], weekly LI-ESWT significantly improved erectile function compared to baseline and was well-tolerated. Similarly, Pedersen et al. [14] conducted a robust sham-controlled RCT, demonstrating significant improvement in IIEF-5 scores and erection quality in the treatment group compared to the sham. In a prospective cohort study by Jang et al. [15], patients receiving LI-ESWT combined with daily tadalafil had higher rates of EHS ≥3 at six months post-treatment compared to those on tadalafil alone. Among retrospective studies, Kohada et al. [16] found long-term functional improvements in patients receiving LI-ESWT post-robot-assisted radical prostatectomy (post-RARP), outperforming both PDE5is alone and non-rehabilitation groups. Frey et al. [17] conducted a single-arm prospective pilot study reporting improvement in IIEF-5 and EHS scores after one year of LI-ESWT, though lacking a control group. Additionally, Karakose and Yitgin [18] and Inoue et al. [20] showed statistically significant erectile function recovery with LI-ESWT when used in combination with PDE5is, although both studies were retrospective and had modest sample sizes. Sholan et al. [19] also compared LI-ESWT monotherapy to LI-ESWT combined with tadalafil in a prospective cohort, reporting greater IIEF-5 recovery in the combination group. Ladegaard et al. [21] performed an RCT with 38 patients and found that LI-ESWT led to statistically significant improvements in IIEF-5 scores and EHS compared to placebo, confirming the benefit in post-radical prostatectomy ED management. 

All studies reported improvements in erectile function post-LI-ESWT, either as monotherapy or in combination with PDE5is. The LI-ESWT protocols varied in energy density (0.09-0.16 mJ/mm²), number of sessions (4-12), and duration of follow-up (ranging from three to 12 months). Outcome measures were largely consistent, with the IIEF-5 being the most commonly used tool, followed by EHS and penile Doppler metrics. Importantly, LI-ESWT was well-tolerated across all trials, with no treatment-related adverse events reported. The quality of evidence varied, with RCTs providing stronger support, while retrospective designs introduced a higher risk of bias. While heterogeneity in intervention protocols, patient characteristics, and follow-up durations precluded meta-analysis, the findings across studies were largely consistent. Taken together, the reviewed evidence supports the safety and potential efficacy of LI-ESWT as part of penile rehabilitation after prostatectomy, especially when initiated early and used alongside pharmacotherapy. However, variation in study designs and small sample sizes underscores the need for larger, high-quality RCTs to determine optimal timing, dosage, and combination strategies. 

Risk of Bias Assessment 

Risk of bias was assessed for each of the 10 included studies using appropriate tools based on study design. RCTs were assessed using the Cochrane RoB 2.0 tool [10] as seen in Table 3, while non-randomised cohort studies were assessed using the NOS [11]. 

**Table 3:** Risk of bias assessment of RCTs using the Cochrane Risk of Bias (RoB) 2.0 tool PDE5i: phosphodiesterase-5 inhibitor; RCTs: randomised controlled trials

Study	Random sequence generation (selection bias)	Allocation concealment (selection bias)	Blinding of participants and personnel (performance bias)	Blinding of outcome assessment (detection bias)	Incomplete outcome data addressed (attrition bias)	Selective reporting (reporting bias)	Other bias
Motil et al. [12]	Low risk	Low risk	High risk (single blind only)	High risk (no mention of outcome assessor blinding)	Low risk	Low risk	Small sample size
Baccaglini et al. [13]	Low risk	Low risk	High risk	High risk	Low risk	Low risk	Unclear
Pedersen et al. [14]	Low risk	Low risk	Low risk (sham-controlled and double-blinded)	Low risk	Low risk	Low risk	None detected
Jang et al. [15]	Low risk	Unclear	High risk (open label)	High risk	Low risk	Low risk	Possible confounding (PDE5i)
Ladegaard et al. [21]	Low risk	Low risk	High risk	High risk	Low risk	Low risk	Unclear

Among the RCTs, Baccaglini et al. [13] was assessed as having a moderate risk of bias. Although randomisation was adequately reported, the absence of participant and personnel blinding introduced a risk of performance bias, which reduced the overall methodological strength. In contrast, Pedersen et al. [14] was judged to have a low risk of bias. This well-designed, sham-controlled RCT incorporated appropriate random sequence generation, allocation concealment, and blinding of both participants and outcome assessors. It also showed minimal attrition and selective reporting bias. Motil et al. [12] was rated as having a low to moderate risk of bias. While the study was only single-blinded, it maintained several features of methodological rigor, including randomisation, an intention-to-treat analysis, and clearly defined primary outcomes. However, its relatively small sample size may have limited the generalisability and statistical power of the findings. Jang et al. [15] was rated as good quality, benefiting from a prospective design, well-matched treatment groups, and adequate follow-up duration. Ladegaard et al. [21] was assessed as having a low risk of bias, due to its strong methodological design, including proper randomisation, blinding, and clear outcome reporting. 

Using the NOS [11] as seen in Table 4, we assessed selection, comparability, and outcome domains. Frey et al. [17] was considered fair quality; although it employed a prospective approach, the absence of a control group and its small sample size limited generalisability. Kohada et al. [16] was also rated as fair quality, with a retrospective design that introduced risks of selection and recall bias. Nevertheless, the study's use of multiple comparator arms and long-term follow-up improved its robustness. Both Karakose and Yitgin [18] and Sholan et al. [19] were evaluated as fair-quality retrospective cohorts. Their non-randomised treatment allocation and limited adjustment for confounding factors contributed to a moderate risk of bias. Inoue et al. [20] was similarly rated as fair quality, with methodological limitations stemming from its retrospective nature and unclear baseline comparability between groups. Overall, out of the 10 studies, three studies were judged to be at low risk of bias, four at moderate risk, and three at higher risk due to retrospective design or lack of control groups. 

**Table 4:** Risk of bias assessment of non-randomised studies using the Newcastle-Ottawa Scale ★: included in the respective study

Study	Adequate definition of cases	Representativeness of cases	Selection of controls	Definition of controls	Control for additional factors	Ascertainment of exposure	Same ascertainment method	Total score (/9)	Quality
Kohada et al. [16]	★	★	★	★	★	★	★	7	Good/fair
Frey et al. [17]	★	–	–	★	–	★	★	4	Fair
Karakose and Yitgin [18]	★	★	–	★	★	★	★	6	Fair
Sholan et al. [19]	★	★	–	★	★	★	★	6	Fair
Inoue et al. [20]	★	★	★	–	–	★	★	5	Fair

Discussion

This systematic review synthesised findings from 10 studies investigating the effectiveness of LI-ESWT for ED following radical prostatectomy. Across diverse study designs, including five RCTs, three retrospective cohort studies, and two prospective cohort studies, LI-ESWT consistently demonstrated a positive impact on erectile function recovery. 

The included RCTs provided the strongest evidence, with Pedersen et al. [14] and Ladegaard et al. [21] showing statistically and clinically significant improvements in IIEF-5 scores compared to sham controls. The third RCT by Motil et al. [12] also revealed early improvements, which were sustained over 12 months, although the results may have been amplified by subsequent pharmacotherapy. These trials highlight the potential of LI-ESWT not only for vascular regeneration but possibly for neuroprotective effects, which are particularly relevant in post-prostatectomy ED where nerve injury is a central mechanism [4,5]. 

Observational studies offered additional insights, particularly regarding the timing, combination therapies, and durability of response. Combination approaches, most notably LI-ESWT with tadalafil, appeared to enhance outcomes, as seen in the studies by Jang et al. [15], Karakose and Yitgin [18], and Sholan et al. [19]. Importantly, none of the studies reported serious adverse events, reinforcing the safety profile of LI-ESWT. 

Nevertheless, several limitations must be acknowledged. Heterogeneity in LI-ESWT protocols (e.g., number of sessions, timing post-surgery, energy settings), variable use of concomitant PDE5is, and differences in follow-up durations precluded quantitative meta-analysis. Moreover, many of the cohort studies were retrospective in nature and lacked control groups, reducing the overall level of evidence. Only three RCTs met the criteria for low risk of bias, while others were subject to selection bias and unmeasured confounding. When considered in the context of the wider LI-ESWT literature, results in general ED populations have often been conflicting, with variability in patient selection, comorbidities, and baseline severity contributing to heterogeneity. By contrast, the post-prostatectomy setting represents a more homogenous cohort in which the primary mechanisms of ED, that is, cavernous nerve injury and impaired penile blood flow, are well-defined. This pathophysiological clarity may explain why the benefits of LI-ESWT appear more consistent and reproducible in post-prostatectomy patients compared to broader vasculogenic or mixed-aetiology populations. 

Despite these limitations, the findings of this review suggest that LI-ESWT is a promising, well-tolerated, and non-invasive option for penile rehabilitation after prostatectomy. It may be most effective when initiated early postoperatively and combined with pharmacotherapy. However, further large-scale, high-quality RCTs are needed to standardise treatment protocols, identify ideal candidates, and determine long-term efficacy and cost-effectiveness. 

Using the Grading of Recommendations Assessment, Development, and Evaluation (GRADE) framework [22], the overall certainty of evidence supporting LI-ESWT for ED following prostatectomy was moderate for improvement in erectile function and high for safety outcomes. The strongest evidence came from RCTs, while observational studies added support for real-world effectiveness, particularly when LI-ESWT was combined with PDE5is. However, the certainty was downgraded for some outcomes due to heterogeneity in treatment protocols, inconsistent follow-up durations, and imprecision in sample sizes. Evidence for long-term functional recovery and combination therapies was graded as low certainty, underscoring the need for additional high-quality trials. Table 5 provides a summary of the GRADE assessment. 

**Table 5:** Grading of Recommendations Assessment, Development, and Evaluation (GRADE) summary IIEF-5: International Index of Erectile Function-5; EHS: Erection Hardness Score; PDE5i: phosphodiesterase-5 inhibitor; RCT: randomised controlled trial; LI-ESWT: low-intensity extracorporeal shock wave therapy

Outcome	No. of studies (design)	Certainty of evidence	Effect estimate (narrative)	Comments
Improvement in erectile function (IIEF-5)	10 studies (5 RCTs, 5 cohort studies)	Moderate	Most studies reported statistically significant improvement in IIEF-5 scores after LI-ESWT, especially in RCTs	Downgraded for inconsistency due to varied protocols and outcome measures
Improvement in erection hardness (EHS ≥3)	6 studies (4 RCTs, 2 cohort studies)	Low	Two studies reported higher rates of EHS ≥3 with LI-ESWT; one showed significance with combined tadalafil use	Downgraded for risk of bias (non-randomised designs) and imprecision
Safety/adverse events	10 studies (all designs)	High	No serious adverse events were reported in any study	Strong consistency across study designs
Long-term functional recovery (≥6 months)	6 studies (2 RCTs, 4 cohort studies)	Low	Several studies showed sustained IIEF-5 improvement at 6-12 months; combination therapies had better outcomes	Downgraded for indirectness and imprecision due to varying follow-up durations
Effect of combination with PDE5i	4 studies (1 RCT, 3 cohort studies)	Low	Combining LI-ESWT with daily tadalafil showed greater improvement in erectile function in all studies	Downgraded for non-randomised comparisons and small sample sizes

## Conclusions

LI-ESWT appears to be a safe and potentially effective therapy for ED in men following radical prostatectomy, particularly when initiated within the first 12 months post-surgery and used alongside PDE5is. However, the evidence remains heterogeneous and limited by methodological weaknesses in many studies. Large-scale, multi-center, double-blind RCTs with standardised protocols and long-term follow-up are needed to establish definitive efficacy, optimal timing, and dosing parameters.

## Appendices

**Table 6:** Search strategy for the databases PMC: PubMed Central

Search strategy	Database used	Number of papers filtered
(("erectile dysfunction"[MeSH Terms] OR "erectile dysfunction"[Title/Abstract] OR "ED"[Title/Abstract]) AND ("prostatectomy"[MeSH Terms] OR "post-prostatectomy"[Title/Abstract] OR "radical prostatectomy"[Title/Abstract]) AND ("shock wave therapy"[MeSH Terms] OR "low-intensity extracorporeal shock wave therapy"[Title/Abstract] OR "LI-ESWT"[Title/Abstract] OR "extracorporeal shockwave"[Title/Abstract]))	PubMed/PMC/MEDLINE	26
(erectile dysfunction) AND (prostatectomy OR post-prostatectomy OR radical prostatectomy) AND ("shock wave therapy" OR "low-intensity extracorporeal shock wave therapy" OR LI-ESWT OR "extracorporeal shockwave")	Cochrane Library	27
(TITLE-ABS-KEY("erectile dysfunction" OR "ED") AND TITLE-ABS-KEY("prostatectomy" OR "post-prostatectomy" OR "radical prostatectomy") AND TITLE-ABS-KEY("shock wave therapy" OR "low-intensity extracorporeal shock wave therapy" OR "LI-ESWT" OR "extracorporeal shockwave"))	Scopus	34
